# Textured Polyester Fiber in Three-Dimensional (3D) Carpet Structure Application: Experimental Characterizations under Compression–Bending–Abrasion–Rubbing Loading

**DOI:** 10.3390/polym15143006

**Published:** 2023-07-11

**Authors:** Gulhan Erdogan, Sinem Yucel, Kadir Bilisik

**Affiliations:** 1 Department of Textile Engineering, Faculty of Engineering, Erciyes University, Talas, Kayseri 38039, Turkey; kadirbilisik@gmail.com; 2 Uniteks Tekstil, Atatürk Organize Sanayi Bölgesi, 10039 sokak, No. 26, Çiğli, İzmir 35620, Turkey; 3 Nanotechnology Application and Research Centre (ERNAM), Erciyes University, Talas9, Kayseri 3803, Turkey

**Keywords:** 3D carpet structure, textured polyester fiber, static loading, abrasion, rubbing, regression model

## Abstract

In this article, textured polyester fiber was used as pile yarn in three-dimensional woven carpet structures. The properties of developed polyester carpets under various mechanical loading were studied. A statistical method was used to analyze the experimental data. Regression models were proposed to explain the relationships between carpet pile height and density. The study showed that the bending rigidity and curvature of dry and wet polyester pile fiber carpets were influenced by pile height and pile density (indirectly weft density) in that the downward concave large bending curvature was obtained from very dense carpet structures. In addition, the average dry bending rigidity of the carpet was over eight times higher than the average wet bending rigidity of the carpet. The thickness loss (%) and resilience (%) for each recovery period of various polyester carpets were proportional depending on the pile density. It was broadly decreased when the pile density was increased due to the compression load carrying capacity per polyester fiber knot, which was higher in carpets having dense knots compared to sparse knots per area. On the other hand, the polyester pile density and height largely affected the carpet mass losses (%) of all textured polyester carpets under an abrasion load. The number of strokes received after completely fractured polyester pile yarns during a rubbing test were increased when the pile heights for each pile density were increased. Findings from the study can be useful for polyester carpet designers and three-dimensional dry or impregnate polyester fiber-based preform designers in particularly complex shape molding part manufacturing.

## 1. Introduction

Polyester carpet can be considered a three-dimensional (3D) structure due to the three yarn sets that are mainly employed as warp for the substrate, including interlaced warp and straight warp (stuffer), filling (interlaced or chain), and pile yarn (Z-yarn). It is called a 3D carpet structure, which is an analogy from 3D preform structures for composite applications in which they have mainly orthogonally interlaced three yarn sets as warp, filling, and z-yarn (binding). One of the main carpet types is a face-to-face carpet structure. It involves four methods: Wilton-type carpet, Brussels-type carpet, Goblen-type carpet, and Axminster carpet. Carpet is one of the most widely used flooring coverings in both residences and workplaces because of its comfort, thermal, sound insulation, and aesthetic properties. Various natural fibers, including protein-based wool, cellulose-based cotton, synthetic-based polyester, polypropylene, acrylic and nylon fibers, or a combination of these materials, can be utilized as pile yarn in carpets [1]. On the other hand, cotton, jute, or a blend of natural fibers in the 3D carpet substrate are commonly used for warp and weft yarns [2].

Poly(ethylene terephthalate) (PET) is the polyester polymer used for fiber production [3]. It is the largest volume of synthetic fiber and over 50 million tons are produced worldwide [4]. Polyester generally has continuous, staple twisted, or crimped yarn forms [5] in use in apparel and interior floor covering textiles such as carpets [6]. On the other hand, recycled polyester from various textile applications is also utilized to make reinforced composites [7]. The critical parameters of polyester fiber are low cost, convenient processability, ease of blending with natural fibers, convenient recyclability, and tailorable performance [8]. Moreover, fiber morphology allows for the balance of dimensional stability, thermal, transport, and mechanical properties [8]. It shows high tenacity and low creep deformation under cyclic loading [9] and good resistance to strain and deformation [6]. It is also identified that fiber cross-section affects its mechanical properties in that full polyester fibers are tough and ductile, whereas hollow fibers are stiffer and more resistant to plastic deformation [10]. Further, it is less fire-resistant and can melt when ignited [11]. In addition, polyester fibers are hydrophobic and do not transport aqueous fluids [6]. In order to increase moisture regain, hydrophilic finishing can be used [12].

Research on carpets is generally aimed at designing carpets for particular end uses and providing guidance for the selection of structural and processing parameters [13,14,15,16]. On the other hand, the characterization of polyester carpets is crucial for identifying their physical, mechanical-thermal, acoustic, appearance retention, and durability properties during service life [17,18,19,20,21,22,23]. For instance, polyester carpet comfort during standing and walking was studied, considering complex biomechanical and psychophysical properties. This could help in the design of more compliant carpets, especially for residential end use [24,25]. Moreover, it was observed that pile length and density were effective for thermal conductivity and sound absorption in polypropylene face-to-face carpets, in which shorter piles and dense loops provided improvement in sound absorption [26,27].

The bending behavior of textile yarn and fabrics, in particular, in polymer-based polyester fibers, was investigated by several researchers to design fabric for apparel and upholstery applications [28,29,30,31,32,33,34,35]. Yarn bending rigidity was obtained from the tensile modulus of its constituent fibers and yarn geometry and structural parameters. It was reported that the yarn bending rigidity decreased as the surface helix angle of yarn and the ratio of tensile-to-shear modulus of the constituent fibers increased [36]. Fabric density and crimp ratio influenced the bending rigidity of single-layer fabric. The flexural rigidities of multi-layered structures depend on the number of fabric layers [37]. Carpet stiffness considering flexural length is an important parameter that is affected by carpet construction, pile density, and pile fiber properties, including fiber type, number of plies, and amount of twist. It was noted that the flexural length of the carpet in the warp and weft after washing decreased significantly [38]. The shifting-point flexure model (SBM) and the fixed-point flexure model (FBM), based on Timoshenko’s elastic theorem, were developed to obtain the flexural rigidity of yarn and fabric structures [39,40]. On the other hand, a polynomial approximation model was proposed to predict the flexural rigidity and flexure length directly via nondimensional parameters as a function of the height-to-length ratio [41].

Carpet structure under a static compression load showed three deformation stages: flexural deformation, mixed deformation including bending and compressive deformations, and compressive deformation in which all fiber piles were affected in the loading zone [42,43]. The effect of structural parameters on face-to-face cut pile carpets under static compression load was studied. It was found that both pile density and pile height influenced the elastic and unrecovered deformation properties of carpet during static loading [44,45,46]. Elastic deformation, especially on polymeric fiber, depended on pile density, whereas unrecovered deformation was mostly related to pile density when the pile height was low [47]. It was demonstrated that through increasing the temperature in the twist heat-setting of air-jet textured polyester pile yarns at the autoclave process, carpet static recovery increased [48,49]. It was experimentally found that carpets with high pile height (16 mm) and high density (3120 piles/dm^2^) exhibited favorable texture retention after static compression loading [50]. It was shown that carpet toughness was highly sensitive towards pile compression resistance and pile interlacement with the substrate, in which pile interlacement may be defined as a function of pile pull-out resistance and friction between yarn sets as well as fiber-to-fiber cohesion [51]. The recovery properties of acrylic polymer carpet after ultraviolet (UV) exposure were investigated. It was shown that UV radiation caused a significant increase in thickness loss [52].

Mathematical models based on probability fracture mechanics considering the Weibull distribution were developed to predict the wear life of wool cut pile carpet [53,54,55]. Generally, models allowed arbitrary initial conditions, life distribution of single fibers, and fatigue site distribution along a fiber for improving fiber properties, carpet constructions, and test methods [56,57,58,59]. Another proposed energy-based model calculated the total energy involved in pile deformation in face-to-face polymeric carpets. This included the bending energy resulting from pile deformation, the energy loss due to sliding pile yarns causing friction, and the energy associated with piles getting jammed at the end of the deformation process. It was found that bending energy was significant compared to frictional and jamming components [60,61]. In their work, Hersh and El-Shiekh (1972) presented a mechanical model to analyze the bending deformation of piles. They focused on calculating the total stored elastic energy associated with bending. They also suggested that considering a non-linear trend for the bending behavior of pile yarns could be reasonable, given the effects of slippage and friction between the fibers within the pile yarns [62]. A novel theoretical approach was introduced for the compression of cut-pile carpets, which relied on the concept of elastic-stored bending energy. The study revealed that the overall energy associated with pile deformation was influenced by both the geometrical and mechanical characteristics of the yarn, as well as the applied compressive load [61,63]. A viscoelastic model based on nonlinear three-element models was applied to the recovery of carpet after static compression loading. It was found that the recovery properties of polyester cut pile yarns was well explained due to plastic deformation as a form of creep, and residual deformation was considered by the model [64,65].

Abrasion is one of the complex loads to degrade the carpet surface during service life, mainly from foot traffic, and it ultimately affects the appearance of the carpet surface due to fiber-to-fiber friction, fiber slippage, and fiber breakages in pile yarns during indoor or outdoor environments [66,67]. The critical parameters related to abrasion can be considered as fiber material and finesses, pile yarn properties including ply number and twist, carpet structural properties such as weave design, interlacement between pile yarn and substrate yarns, and out-of-plane pile length and density [68,69]. The abrasion behavior of carpets is collectively influenced by fiber, yarn, and carpet constructional parameters [2]. Onder and Berkalp (2001) found that abrasion resistance of face-to-face acrylic/wool/polypropylene blended carpet was related to pile yarn and carpet structural parameters considering substrate (warp, weft, and stuffer), pile height, and pile density [70]. Another study was carried out on the abrasion resistance of flocked fabric by Bilisik and Yolacan (2009). It was obtained that the flock fiber density and fiber length influenced the abrasion properties of laminated flock fabric, in which dense and short polyamide flock fiber showed better abrasion resistance due to large fiber surface area and flexure rigidity [71]. It was also obtained that the abrasion loss increased with an increase in fiber diameter [72].

Token rubbing test on flocked fabrics exhibited that their warp and weft tearing strengths in a wet state were slightly higher than those of the dry state due to the existence of cotton fiber and the lubrication effect of acrylic adhesive in fabrics [73]. As the stroke number increased, there was an ordinary decrease in the warp and weft directional tensile strength and an elongation of flocked fabric. Moreover, the stroke number of a flocked fabric in its wet form, compared to its dry form, exhibited lower values. This can be attributed to the poor wet properties of the acrylic adhesive, which was the main factor responsible for this effect [74]. Furthermore, polyamide (nylon) pile fiber carpet inkjet printing with printing ink with a low thickener concentration has good rubbing fastness and color yield [75].

The purpose of this study was to experimentally determine the static loading, bending rigidity, abrasion, and rubbing properties of textured polyester woven carpet structures. Moreover, generated experimental data were also statistically analyzed for possible improvement on the design of carpet structures.

## 2. Materials and Methods

### 2.1. Carpet Structure

**Figure 1:**
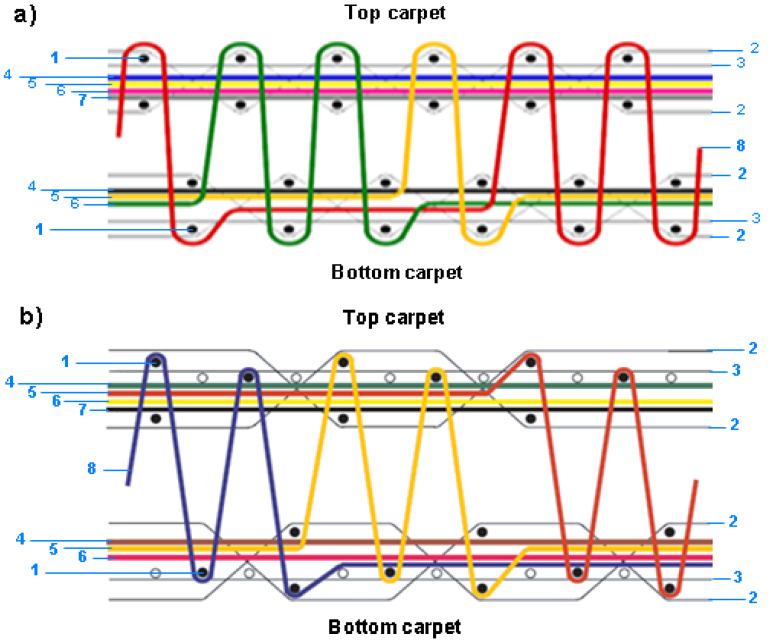
Schematic views of carpet structures. (**a**) The 2/2 V weave design: (1) weft, (2) warp (interlace or chain), (3) warp (stuffer), (4–8) pile yarns [76]. (**b**) The 1 + 2/3 V weave design: (1) weft, (2) warp (interlace or chain), (3) warp (stuffer), (4–8) pile yarns [76].

Polyester carpet samples were obtained from Gumussuyu Halı Inc. (Kayseri, Turkey) subsidiary of Erciyes Anadolu Holding (Kayseri, Turkey). Carpets were fabricated using the Wilton face-to-face carpet weaving principle on a Van De Wiele carpet loom with three rapiers. They were designed using the Weaving and Booria programs compatible with the loom. Carpet samples were made as 2/2 V and 1 + 2/3 V weave constructions. They are illustrated in Figure 1a,b. Both polyester woven carpet structures have noninterlaced stuffer and interlaced warp (80/20% polyester/cotton blended yarn), weft (100% jute), and colored pile yarns (100% polyester). Table 1 presents specifications of the fiber, yarn, and substrate properties of the carpets.

Three different density carpet samples were made as 48 warp × 48 weft (loose), 48 warp × 55 weft (dense), and 48 warp × 70 weft (very dense) ends/10 cm. Each of them were made with three distinct pile heights at 3.34–3.54 mm (short), 4.21–4.83 mm (medium), and 5.88–6.46 mm (long). In addition, loose and medium density carpet structures were made with a 2/2 V weave pattern, whereas dense carpet was constructed using a 1 + 2/3 V pattern. The pile yarn in each carpet was textured polyester fiber and its yarn linear density was 165 tex. It was composed of a continuous bundle of filaments and had no twist. Pile weight and carpet weight varied between 954–1981 g/m^2^ and 1797–2877 g/m^2^, respectively. Measured pile height and carpet thickness were varied: 3.34–6.46 mm and 5.91–9.65 mm, respectively. Table 2 shows polyester pile yarn and carpet structure properties. All data generated on carpet samples were average values.

#### 2.1.1. Bending (Flexure) Test

**Figure 2:**
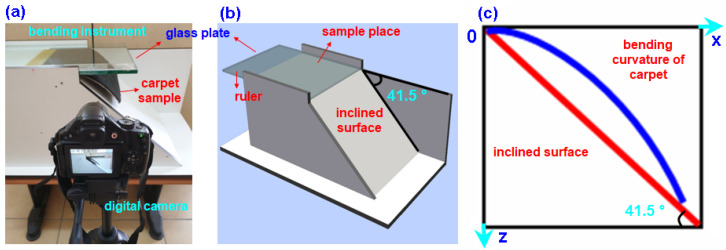
(**a**) Bending rigidity instrument during testing of carpet structure in warp direction, (**b**) schematic view of bending rigidity instrument, and (**c**) bending curvature obtained from digital camera during testing.

A modified bending test instrument was used based on the fixed-angle flexometer method as explained in ISO 4604 test standards [77]. The fixed-angle flexometer method or inclined plane method principally originated from the Peirce cantilever test [78,79]. The bending angle in the cantilever base test devise was θ = 41.5°. The fabric sample was placed on a smooth horizontal platform, where one end of the sample was aligned with the edge of the platform. A glass plate was positioned on top of the fabric sample, aligning the zero point of the slide rule with the starting point. The slide rule was gradually moved to facilitate the bending of the sample under its own weight. The bending test was performed until the sample made contact with the inclined platform at its end. At the conclusion of the test, the bending length was determined through measuring from the starting point on the slide rule. Figure 2a–c depicts the bending test instrument utilized during the bending test. The sample sizes of carpet were 2.5 cm (width) × 15 cm (length) for apparel fabric [80,81] and 30 cm (width) × 30 cm (length) for technical fabric [77]. Warp directional flexural tests on carpet samples were conducted for dry and wet forms and repeated four times.

A digital camera (CANON PowerShot SX30 IS, Tokyo, Japan) was integrated with an in-house-developed flexure test instrument. It recorded the sample curvature end of the testing (Figure 2a) and the image was uploaded to the SnagIt drawing program to find the bending curvature (Figure 2c) [82]. The bending curvature regression equations were determined using the image received from the drawing program. It was computed within MATLAB R2016a [83]. This was achieved using MATLAB’s numerical integration and standard plotting tools. Bending length and bending rigidity for apparel and technical fabrics were computed following Equations (1)–(3).

(1) $$c=\frac{l}{2}$$

(2) $$G_{1}=0.1\times m\times c^{3}$$

(3) $$G_{2}=9.81\times m\times {\left(\frac{l}{2}\right)}^{3}$$

where m is the fabric unit areal weight (g.m^−2^); l is the fabric length of overhang (apparel fabric, cm; technical fabric, m); c is the bending length (cm, m); G_1_ and G_2_ are the bending rigidity of apparel (mg.cm) and technical fabrics (mN.m), respectively; 9.81 is the gravitational constant (m.s^−2^).

#### 2.1.2. Static Loading Test

**Figure 3:**
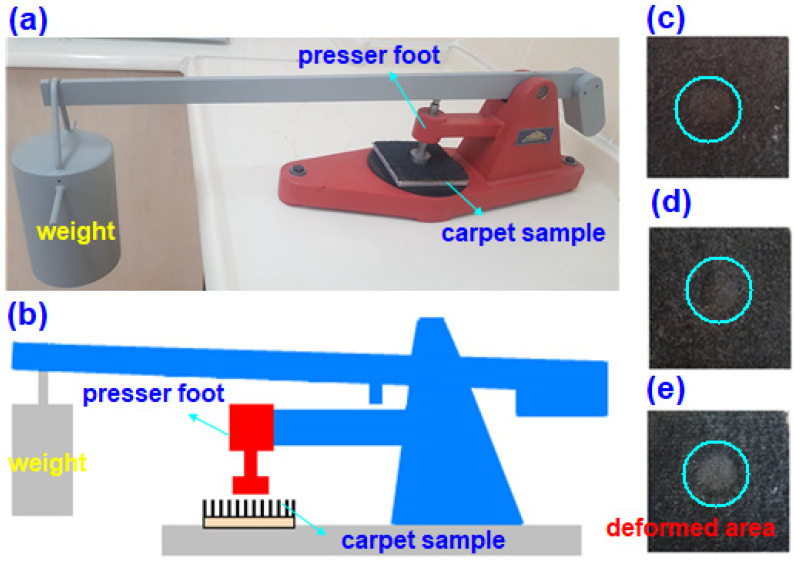
(**a**) Static loading instrument during testing of carpet structure, (**b**) schematic view of static loading instrument with carpet sample, (**c**) image of deformed loose carpet long pile (IPES12), (**d**) image of deformed dense carpet long pile (2PES12), and (**e**) image of deformed very dense carpet long pile (3PES12) structure samples (digital photos).

A static loading test on face-to-face woven carpet samples was ordinarily carried out following BS 4939 [84] and ISO 3416 [85] test standards. The sample sizes of carpet for static loading were 10 cm × 10 cm. Before starting the test, sample initial thickness (h_0_) was measured. Later, it was uploaded in the test instrument where pressure on the sample was applied (7 kg/cm^2^, 0.687 MPa) via dead weight (10 kg). The samples under static loading were held 24 h (h). After that, the thickness loss on the sample was measured according to short (2 min and 1 h), medium (1 day and 3 days), and long (3 years) time periods. The test was repeated twice due to limited samples. Figure 3a–e shows the actual and schematic static loading instrument during testing of the carpet structure, and failed carpet samples.

**Figure 4:**
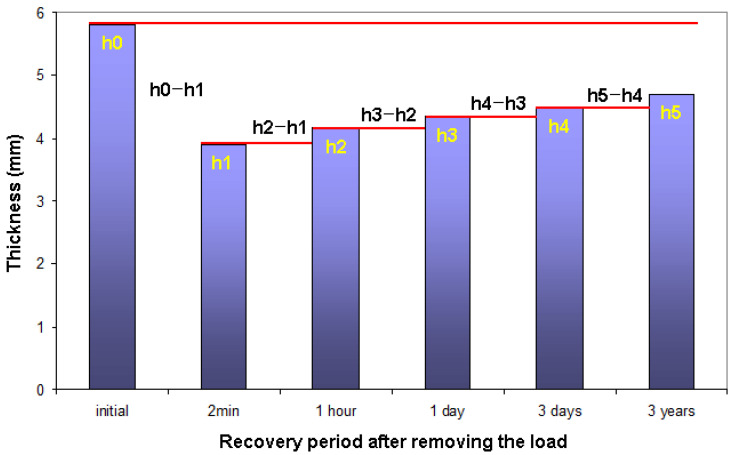
Recovery period and thickness relations after static loading on carpet samples.

The thickness loss and resilience of the carpet after prolonged heavy static load were determined using the following Equations (4)–(13). The relations are also presented in graphical form as exhibited in Figure 4 [46].

(4) $${\mathrm{TL}}_{2\min}\;(\%)=\left(\frac{h_{0}-h_{1}}{h_{0}}\right)\times 100$$

(5) $${\mathrm{TL}}_{1h}(\%)=\left(\frac{h_{0}-h_{2}}{h_{0}}\right)\times 100$$

(6) $${\mathrm{TL}}_{1d}(\%)=\left(\frac{h_{0}-h_{3}}{h_{0}}\right)\times 100$$

(7) $${\mathrm{TL}}_{3d}(\%)=\left(\frac{h_{0}-h_{4}}{h_{0}}\right)\times 100$$

(8) $${\mathrm{TL}}_{3y}(\%)=\left(\frac{h_{0}-h_{5}}{h_{0}}\right)\times 100$$

(9) $$R_{}\;(\%)=\left(\frac{h-h_{1}}{h_{0}-h_{1}}\right)\times 100$$

(10) $$R_{1h}(\%)=\left(\frac{h_{2}-h_{1}}{h_{0}-h_{1}}\right)\times 100$$

(11) $$R_{1d}(\%)=\left(\frac{h_{3}-h_{1}}{h_{0}-h_{1}}\right)\times 100$$

(12) $$R_{3d}(\%)=\left(\frac{h_{4}-h_{1}}{h_{0}-h_{1}}\right)\times 100$$

(13) $$R_{3y}(\%)=\left(\frac{h_{5}-h_{1}}{h_{0}-h_{1}}\right)\times 100$$

where h_0_ is the initial thickness (mm), h_1_ is the thickness after 24 h compression (after 2 min recovery time) (mm), h_2_ is the thickness after 1 h recovery time, h_3_ is the thickness after 1 day (24 h) recovery time, h_4_ is the thickness after 3 days recovery time, h_5_ is the thickness after 3 years recovery time, TL is the thickness loss (%), TL_1h_ is the thickness loss after 1 h (%), TL_1d_ is the thickness loss after 1 day (%), TL_3d_ is the thickness loss after 3 days (%), TL_3y_ is the thickness loss after 3 years (%), R is the resilience (%), R_1h_ is the resilience after 1 h, R_1d_ is the resilience after 1 day (24 h), R_3d_ is the resilience after 3 days, and R_3y_ is the resilience after 3 years.

#### 2.1.3. Abrasion (Martindale) Test

**Figure 5:**
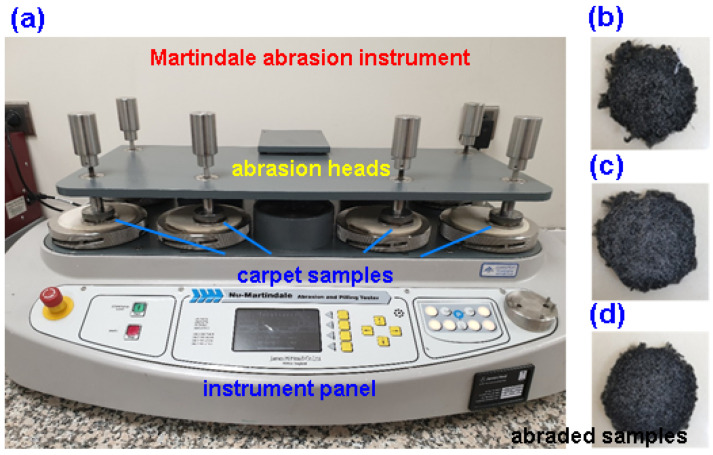
(**a**) Martindale abrasion instrument during testing of carpet structure, (**b**) image of abraded loose carpet long pile (IPES12), (**c**) image of abraded dense carpet long pile (2PES12), and (**d**) image of abraded very dense carpet long pile (3PES12) structure samples (digital photos).

The abrasion properties of the polyester carpet structures were determined using the Martindale abrasion test method (ISO 12947-3) [86]. A Nu-Martindale Abrasion Test instrument (James H Heal, Halifax, UK) was used to evaluate the mass loss of carpet after abrasion in compliance with the TS EN ISO 12947-3 standards, as shown in Figure 5a–d. Mass loss values were recorded at the end of each 5000-, 10,000-, 20,000-, 30,000-, 40,000-, and 50,000-abrasion cycle. Standard wool fabric was used for abrasion and the pile surface of the carpet samples were abraded under pressure (12 KPa). Thickness measurements were also performed, since the carpet sample had a thickness loss tendency after abrasion cycles, using a thickness gauge (Elastocon EV 07, Bramhult, Sweden) [71]. The experiment was repeated twice due to limited test materials.

#### 2.1.4. Rubbing (Token) Test

**Figure 6:**
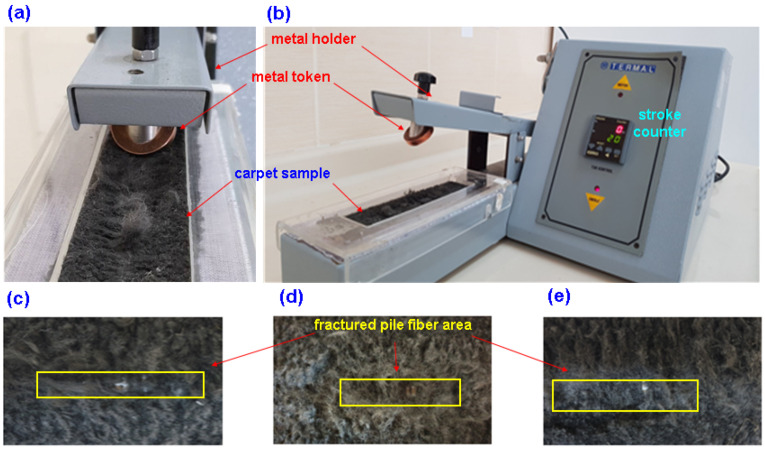
Crockmeter with metal token holder during rubbing test on carpet sample. (**a**) Front view, (**b**) complete view of instrument, (**c**) image of fractured loose carpet long pile (IPES12), (**d**) image of fractured dense carpet long pile (2PES12), and (**e**) image of fractured very dense carpet long pile (3PES12) structure samples (digital photos).

To assess the rubbing behavior of the carpet sample according to the BS 2543 test standards, a crockmeter (Termal, Istanbul, Turkey) equipped with a metal token holder was employed [87]. The dry carpet’s resistance to linear rubbing was evaluated using a metal token holder positioned at a 45° angle to the carpet pile surface. The areas of the carpet pile that exhibited deformations were identified through a visual assessment based on images captured using a digital camera. The dry carpet’s resistance to linear rubbing was evaluated using a metal token holder positioned at a 45° angle to the carpet pile surface. The metal token utilized in the study had a diameter of approximately 29 mm, a thickness of 6 mm, and was made of copper [71]. The token rubbing test was employed to simulate carpet hand cleaning. In this method, a rectangular specimen was positioned on the rubbing area of the crockmeter, and a metal token holder, inclined at a 45° angle to the carpet pile surface, was used to rub the specimen under low pressure (9 N). Figure 6a–e show the token rubbing test instrument during the rubbing test on a carpet sample. The token rubbing test was conducted until a visually consistent level of deformation was observed across all samples. The results of the test were expressed in terms of the stroke number. In the context of this study, a stroke was defined as a single back-and-forth movement of the metal token holder along the surface of the carpet pile.

The neat carpet sample and polyester pile mass loss of weight measurements were performed based on TS 251 [88] using an Ohaus Adventurer^TM^ Pro AV812 (Ohaus Corp., Parsippany, NJ, USA) digital balance. The error in the measurement of weight was ±0.1 mg. The neat pile thickness, pile thickness after static loading and Martindale tests, and the neat carpet thickness were measured based on TS 7125 [89] and TS 3374 [90] using an Elastocon EV07 digital device, respectively. All mechanical tests were conducted in a standard laboratory atmosphere having a temperature of 23 °C ± 2 °C and relative humidity of 50 ± 10% [91]. A high-resolution digital camera (CANON PowerShot SX30 IS, Tokyo, Japan) was used to image the damaged surface of carpet samples after static loading and Martindale abrasion and token rubbing tests.

### 2.2. Statistical Model

Statistical modelling of some of the static loading (thickness loss and resilience) and abrasion (thickness loss) data on carpet samples was developed using “Design Expert” software. The best models on carpet were obtained, and the corresponding regression equations and regression curves were fitted. The analysis of variance (ANOVA) tables for static loading (thickness loss and resilience) and abrasion property, specifically the thickness loss, were measured, and the significance of the models was determined using *p*-values smaller than 0.05. The ANOVA tables revealed significant interactions between the pile height (A) and density (B) in both the static loading and abrasion tests. These interactions were taken into account when developing the regression equations. The generated data were subjected to a normality test, which indicated that the data exhibited a distribution that was approximately aligned with the normality line and conformed to a normal distribution.

## 3. Results and Discussion

### 3.1. Carpet Structure Bending Results

**Figure 7:**
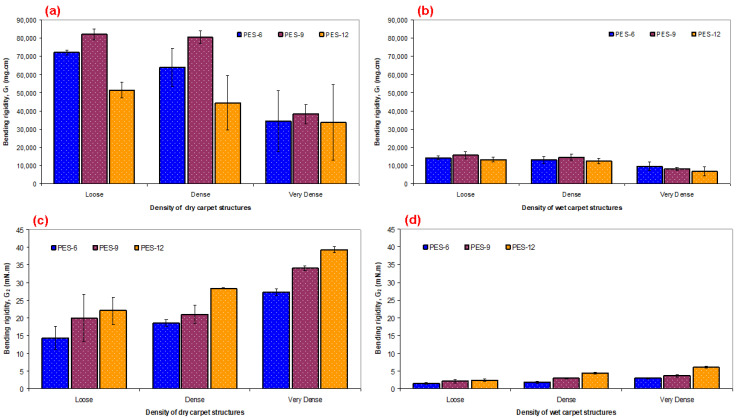
Flexural results on dry and wet forms of carpet samples. (**a**,**c**) Dry carpet samples for apparel and technical fabric methods, respectively, and (**b**,**d**) wet carpet samples for apparel and technical fabric methods, respectively.

Bending rigidity results on various carpet structures are presented in Table 3 and Figure 7a–d for both apparel fabric and technical fabric bending test methods. As shown in Table 3 and Figure 7a–b, the dry bending rigidity based on the apparel fabric test method of 2PES6-G_1_ and 3PES6-G_1_ was decreased by 12.67% and 108.63% compared to 1PES6-G_1_, respectively. Similar changes were obtained from remaining dry polyester carpet structures including 1PES9, 2PES9, 3PES9, 1PES12, 2PES12, and 3PES12 (Figure 7a). On the other hand, the polyester pile height probably indirectly affected the bending rigidity of carpet in that carpet areal density was incrementally increased when the pile height increased. The wet bending rigidity of 2PES6-G_1_ and 3PES6-G_1_ was decreased by 9.74% and 47.89% compared to 1PES6-G_1_, respectively. Similar trends were obtained from remaining wet carpet structures including 1PES9, 2PES9, 3PES9, 1PES12, 2PES12, and 3PES12 (Figure 7b). This indicated that the bending rigidity of polyester carpet samples from dry and wet cases exhibited similar results. The results from bending for apparel fabric showed that when the carpet weight (areal density, g/m^2^) increased, the bending rigidity (mg.cm) decreased from loose to very dense carpet structures due to the increase in weft density. Moreover, the average dry bending rigidity of polyester carpet samples was 4.61 times higher than the average wet bending rigidity of samples due to water uptake increasing the carpet weight.

As shown in Table 3 and Figure 7c,d, the dry bending rigidity, based on the technical fabric test method, increased by 21.95% and 47.26% for 2PES6-G2 and 3PES6-G2, respectively, compared to 1PES6-G2. Similar changes were observed in the remaining dry polyester carpet structures, including 1PES9, 2PES9, 3PES9, as well as 1PES12, 2PES12, and 3PES12 (Figure 7c). On the other hand, it is likely that the pile height indirectly influenced the bending rigidity of the carpet, as the carpet’s areal density increased incrementally with higher pile heights. The wet bending rigidities of 2PES6-G2 and 3PES6-G2 were increased by 18.78% and 50.34% compared to 1PES6-G1, respectively. Similar trends were nearly achieved from remaining wet carpet structures including 1PES9, 2PES9, 3PES9, and 1PES12, 2PES12, and 3PES12 (Figure 7d). This indicates that the bending rigidity of polyester carpet samples showed similar results in both dry and wet conditions. The bending results for technical fabric showed that as the carpet weight (areal density, g/m^2^) increased, the bending rigidity (mN.m) also increased, moving from loose to very dense carpet structures. This increase could be attributed to the larger sample size used for bending tests in technical fabric, with a sample width 12 times higher compared to the sample size used for bending tests in apparel fabric, where the sample size affects the bending stiffness. Furthermore, the average dry bending rigidity of polyester carpet samples was 8.11 times higher than the average wet bending rigidity of carpet samples, attributed to the increase in the weight of wet carpets. For future studies, the bending test for technical fabric can be simplified to determine the flexural properties of heavy three-dimensional dry or impregnated polymeric preforms, especially in complex shape molding for manufacturing specific parts.

#### Carpet Structure Curvature Results

**Figure 8:**
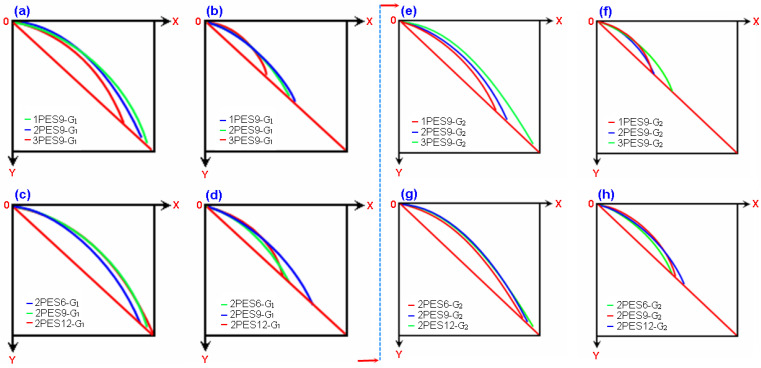
Bending curvature results on carpet depending on apparel fabric and technical fabric bending test principles. (**a**,**e**) Various densities of dry carpets (medium pile height), (**b**,**f**) various densities of wet carpets (medium pile height), (**c**,**g**) dense dry carpets (various pile height), and (**d**,**h**) dense wet carpet samples (various pile height).

Bending curvature results on various dry and wet carpets depending on apparel fabric and technical fabric bending test principles are illustrated in Figure 8a–h. Regression equations of bending curvature results on various dry and wet carpet structures are presented in Table 4. As shown in Table 3 and Figure 8a–h, the overhang lengths based on the apparel fabric test method of dry 2PES9-G_1_ and 3PES9-G_1_ carpet samples were decreased by 0.35% and 35.41% compared to 1PES9-G_1_, respectively. A similar tendency was found in the remaining dry carpet structures including 1PES6, 2PES6, 3PES6, and 1PES12, 2PES12, and 3PES12 (Table 3, Figure 8c). However, the pile height probably insignificantly affected the bending curvature of carpet in that carpet areal density was incrementally increased when the pile height increased (Figure 8c). The overhang lengths of wet 2PES9-G_1_ and 3PES9-G_1_ carpet samples were decreased by 2.2% and 29.92% compared to 1PES9-G_1_, respectively (Figure 8b). Nearly the same trends were obtained from rest of the wet polyester carpet structures including 1PES6, 2PES6, 3PES6, 1PES12, 2PES12, and 3PES12 (Table 3, Figure 8d). This indicated that the bending curvature of carpets from dry and wet cases illustrated similar results. The results from bending curvature for the apparel fabric test showed that when the carpet weight (areal density, g/m^2^) increased, the bending curvature (cm) decreased from loose to very dense carpet structures due to the increase in weft density. In addition, downward concave small bending curvature was obtained from very dense carpet structures. Further, the average dry bending curvature of carpet samples was 39.33% higher than the average wet bending curvature of carpet samples due to the increase in wet polyester carpet weight. Thus, large bending curvatures from all dry carpets were obtained compared to the wet carpets (Figure 8a–d).

As shown in Table 3 and Figure 8a–h, the overhang lengths based on the technical fabric test method of dry 2PES9-G_2_ and 3PES9-G_2_ carpets were increased by 1.99% and 12.05% compared to 1PES9-G_2_, respectively. Similar results were found from remaining dry carpet structures including 1PES6, 2PES6, 3PES6, 1PES12, 2PES12, 3PES12 (Table 3, Figure 8e). However, it is likely that the pile height had a minimal effect on the bending curvature of the carpet, as the carpet’s areal density incrementally increased with higher pile heights (see Figure 8g). Compared to 1PES9-G2, the overhang lengths of wet 2PES9-G2 and 3PES9-G2 carpets exhibited increases of 10.58% and 12.26%, respectively (refer to Figure 8f). Similar trends were observed in the remaining wet carpet structures, including 1PES6, 2PES6, 3PES6, as well as 1PES12, 2PES12, and 3PES12 (see Table 3 and Figure 7h). This suggests that the bending curvature of carpets in both dry and wet conditions exhibited similar results. The results of the bending curvature test for technical fabric showed that as the carpet weight (areal density, g/m^2^) increased, the bending curvature (cm) also increased, transitioning from loose to very dense carpet structures. This increase can be attributed to the higher weft density and the larger sample size used in the bending test for technical fabric, where the sample width was 12 times greater compared to the bending test for apparel fabric, where the sample size affects the bending curvature. In addition, loose carpet structures exhibited a downward concave small bending curvature, while very dense carpet structures exhibited a downward concave large bending curvature. Moreover, the average dry bending curvature of carpets was more than twice as high as the average wet bending curvature of carpets, attributed to the increase in wet carpet weight. Thus, it was found that all dry carpets exhibited larger bending curvatures compared to the wet carpets (see Figure 8e–h). It was concluded that bending via the technical fabric test can simplify for the determination of heavy three-dimensional dry or impregnate polymeric preforms’ flexural properties, particularly for complex shape molding part manufacturing.

Regression equations of the bending curvature of the dry and wet form of carpets based on the apparel fabric and technical fabric bending tests were obtained using MATLAB R2016a [83]. This was achieved using MATLAB’s numerical integration and standard plotting tools. Regression equations obeyed the polynomial function where n varied between 1.7630–0.7361 for apparel fabric and 0.9925–0.7570 for technical fabric. The coefficients of regression on the bending apparel and technical fabric test data were between 0.9999 and 0.9524, which was considered well fitted for the measured values (Table 4).

### 3.2. Carpet Structure Static Loading Results

**Figure 9:**
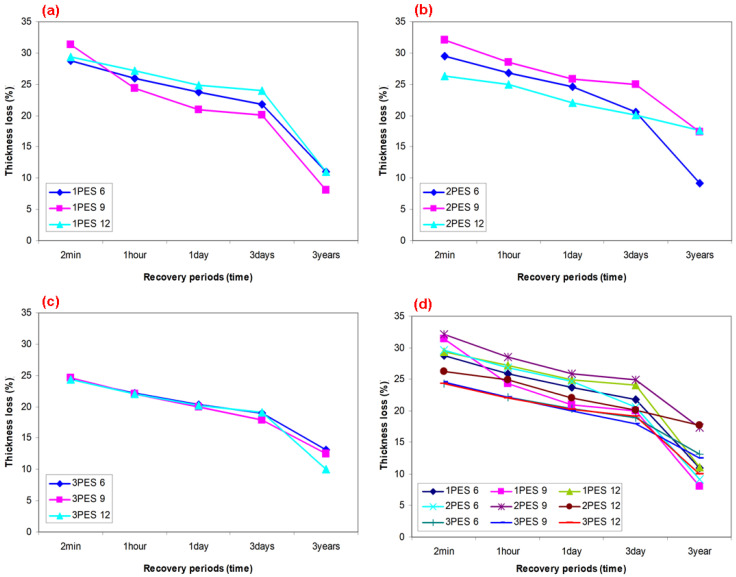
Various recovery period and thickness and thickness loss relations after static loading on the dry carpets. (**a**) Loose dry carpet (various pile height), (**b**) dense dry carpet (various pile height), (**c**) very dense dry carpet (various pile height), and (**d**) various densities and pile heights of dry carpet samples.

**Figure 10:**
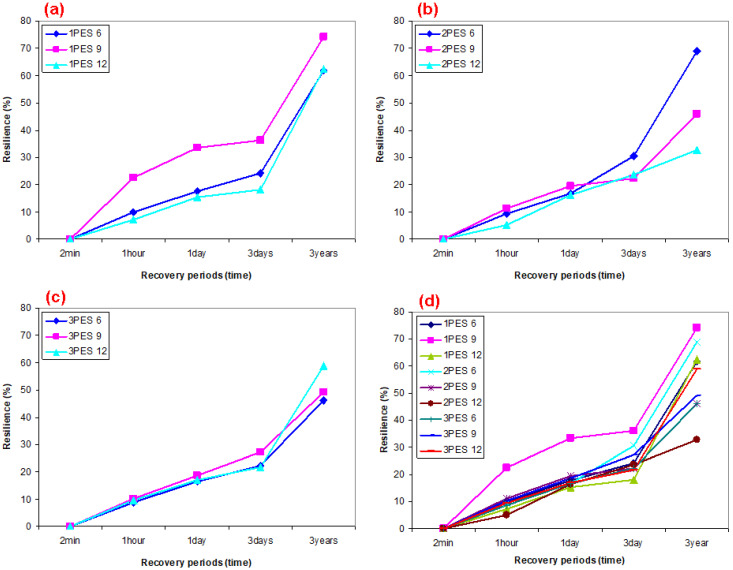
Various recovery period and resilience relations after static loading on the dry carpets. (**a**) loose dry carpet (various pile height), (**b**) dense dry carpet (various pile height), (**c**) very dense dry carpet (various pile height), (**d**) various densities and pile heights of dry carpet samples.

Static loading (compression) results on the thickness (mm), thickness loss (%), and resilience (%) of various polyester pile carpet structures are presented in Table 5 and are illustrated in Figure 9a–d. In addition, Figure 10a–d illustrates various recovery period and resilience relations after static loading on dry carpet samples.

As shown in Table 5 and Figure 9a–d, the carpet thickness loss (%) of various recovery periods of all carpets almost linearly decreased. The average dry carpet thickness loss (%) under a vertical distributed load (compression) for various recovery periods including after 2 min, 1 h, 1 day, and 3 years of 2PES6 and 3PES6 were decreased by 0.45% and 12.53% compared to 1PES6, respectively. Similar results were roughly obtained from rest of the carpet structures including 1PES9, 2PES9, 3PES9, 1PES12, 2PES12, 3PES12, where when the pile density increases, the variation of thickness loss decreases for all polyester pile heights (Figure 9a–c). This is perhaps related to the increase in knots density, which affected polyester fiber-to-fiber friction (cohesion friction) and indirectly weft density. On the other hand, the thickness loss for each recovery period from loose to very dense carpets proportionally depended on the pile density. In general, it decreases as the pile density increases due to the compression load carrying capacity per knot. This capacity is higher in carpets with dense knots compared to those with sparse knots per unit area. In addition, the effect of pile heights on the carpet thickness loss for each recovery period was hardly significant from loose to very dense polyester carpets because of complex buckled pile yarn deformation mechanism under the constrained substrate, where critical structural parameters were considered as pile yarn specifications including linear density, knot density, polyester fiber-to-fiber friction, and twisted plied or untwisted textured forms [92].

As shown in Table 5 and Figure 10a–d, the carpet resilience (ability to recover from pile deformation or gain from total thickness loss, %) of various recovery periods of all carpets sharply increased. The average resilience (%) under static load (compression) for various recovery periods including after 2 min, 1 h, 1 day, and 3 years of 2PES6 and 3PES6 slightly decreased compared to 1PES6. Similar results were obtained from the remaining carpet structures, wherein an increase in pile density resulted in a decrease in resilience variation across all pile heights (refer to Figure 10a–c). This may be attributed to the increased density of knots and the structural weft density. On the other hand, the resilience for each recovery period from loose to very dense carpets depended on the pile density. Generally, it decreases as the pile density increases due to the strong resistance generated by carpets with dense knots per unit area. Furthermore, the impact of pile heights on the resilience of polyester carpet became marginally significant for each recovery period. As the pile height increased from loose to very dense carpets, the resilience of the carpet tended to decrease. This could be attributed to the time-dependent deformation mechanism of the polyester pile yarn under the constrained substrate, as well as the presence of complex residual stress and stress relaxation. These critical structural parameters include pile yarn specifications such as linear density, knots density, fiber-to-fiber friction, and the choice between twisted plied or untwisted textured forms. We plan to conduct further research on these aspects through generating load–displacement and stress–strain curves to elucidate the critical parameters of the carpet.

### 3.3. Martindale Abrasion Results

**Figure 11:**
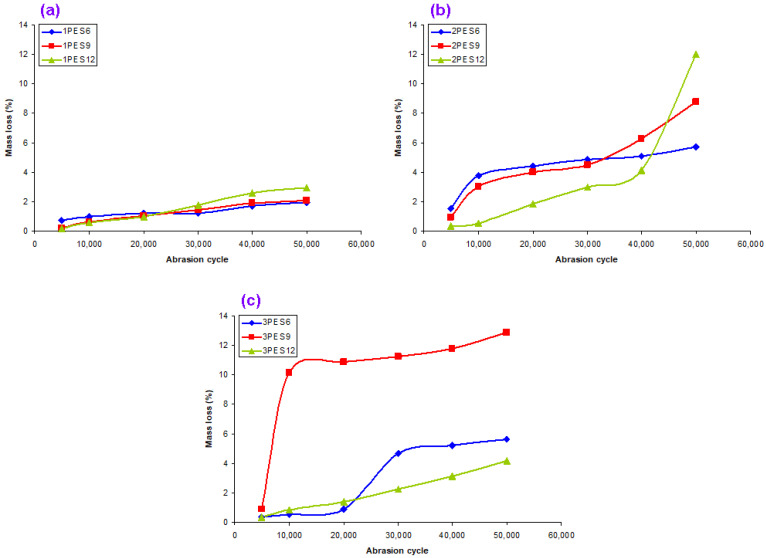
Various abrasion cycles and pile mass loss (%) relations after a Martindale abrasion test on dry carpets. (**a**) Mass loss (%) of loose carpets (various pile height), (**b**) mass loss (%) of dense carpets (various pile height), and (**c**) mass loss (%) of very dense carpets (various pile height).

**Figure 12:**
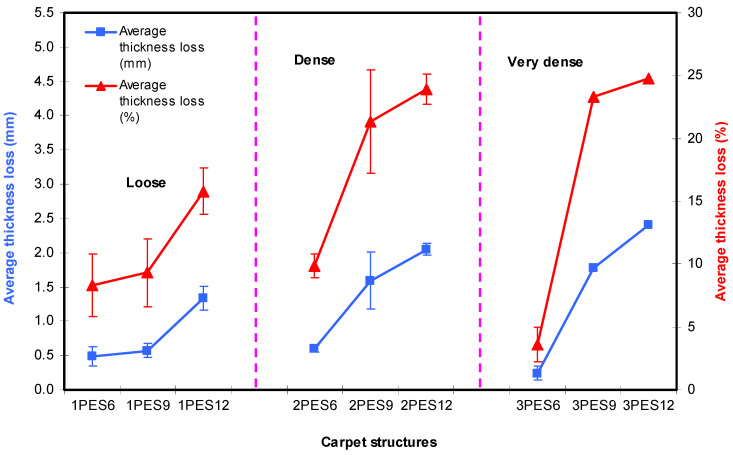
Abrasion cycle (50,000) and carpet thickness loss (mm, %) relations after a Martindale abrasion test on dry carpet samples.

Martindale abrasion results on carpet structure for different abrasion cycles, pile mass loss and thickness loss is presented in Table 6. Figure 11a–c shows various abrasion cycles and pile mass loss (%) relations after a Martindale abrasion test on the dry carpet samples. Furthermore, Figure 12 exhibits abrasion cycle (50,000) and carpet thickness loss (mm, %) relations after testing on carpets.

As shown in Table 6 and Figure 11a–c, when the pile density increased, the carpet mass losses (%) of all carpets increased. For instance, the average carpet mass losses (%) of 1PES9, 2PES9, and 3PES9 carpet samples were 1.23%, 4.59%, and 9.63% higher, respectively. This is because of the number of knots per area. At the same pile density, when the pile height increased, the carpet mass losses (%) of all carpets were nearly increased. For instance, the average carpet mass losses (%) of 1PES6, 2PES9, and 3PES12 carpets were 1.31%, 1.23%, and 1.52% raised, respectively. On the other hand, when the abrasion cycles were increased from 5000 cycles to 50,000 cycles, the average carpet mass losses (%) increased from 0.63% to 6.25%. Moreover, it was identified that the effect of substrate architecture on the carpet abrasion properties was insignificant.

As shown in Table 6 and Figure 12, when the pile density increased, the carpet thickness losses (%) of all carpets were mainly increased after 50,000 abrasion cycles due to more out-of-plane fibers being in contact at the abraded area. For example, the average carpet thickness losses (%) of 1PES9, 2PES9, and 3PES9 carpet samples increased by 9.33%, 21.32%, and 23.30% compared to the initial carpet thickness values, respectively. At the same pile density, when the pile height increased, the carpet mass losses (%) of all carpet samples were nearly increased. Similarly, the average carpet thickness losses (%) of 1PES6, 1PES9, and 1PES12 carpet samples were 8.30%, 9.33%, and 15.81% greater, respectively.

### 3.4. Token Rubbing Results

**Figure 13:**
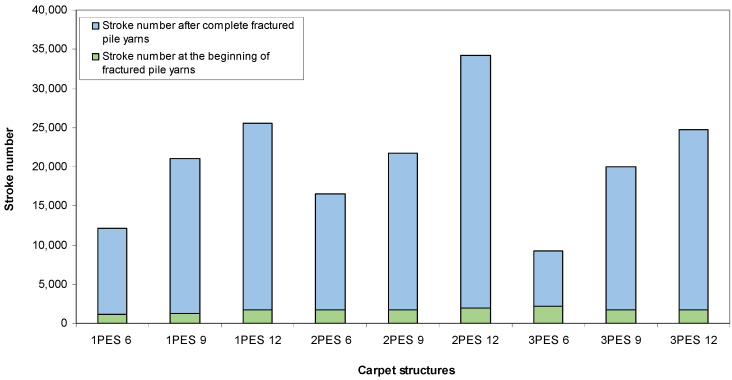
Token rubbing test results for various dry carpets.

Token rubbing test results on carpet samples are presented in Table 7. Figure 13 exhibits token rubbing test results for various dry carpets. As shown in Table 7 and Figure 13, the number of strokes after the complete fracture of pile yarns increased as the pile heights for each pile density were increased. Comparable relationships were observed in both loose and dense carpet structures, with the exception of very dense carpet structures. Furthermore, it was discovered that the number of strokes after the complete fracture of pile yarns and the number of strokes at the onset of fractured pile yarns were proportional for almost all carpets.

### 3.5. Statistical Modelling Results

**Figure 14:**
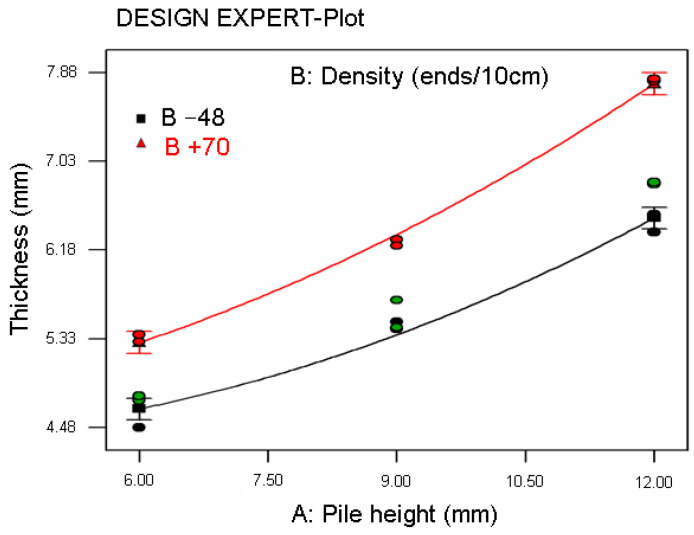
Relationship between carpet thickness and pile height after 3-day time period of static loading for various carpet densities.

**Figure 15:**
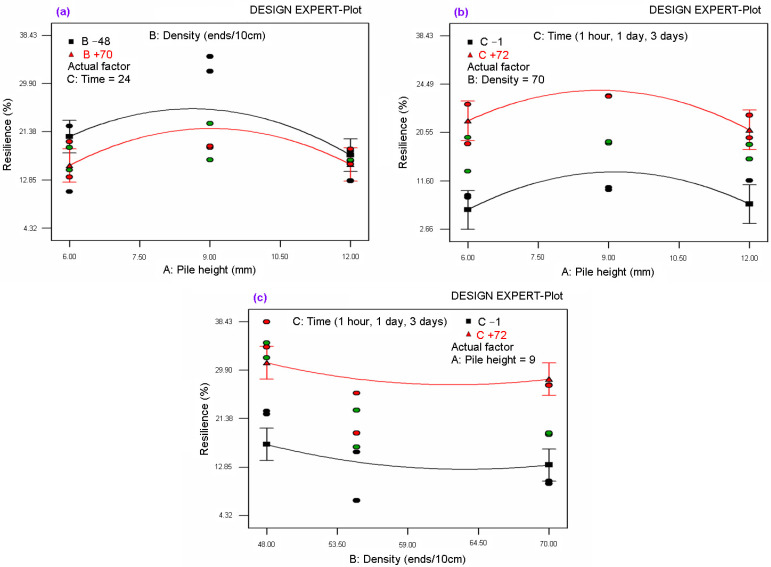
(**a**) Relationship between resilience and pile height after 1-day time period of static loading for various carpet densities; (**b**) relationship between resilience and pile height after 1 h, 1-day, and 3-day time period of static loading for very dense carpet; and (**c**) relationship between resilience and carpet density after 1 h, 1-day, and 3-day time period of static loading for medium pile height.

**Figure 16:**
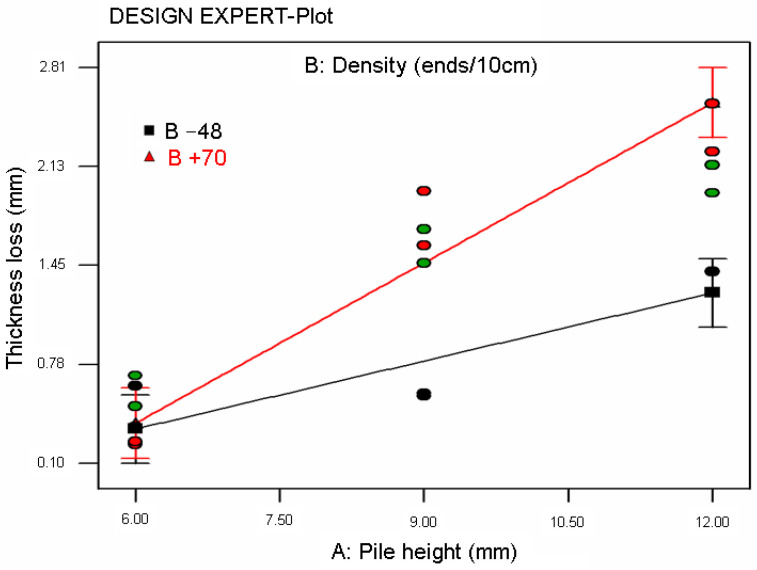
Relationship between carpet thickness loss and pile height after abrasion test for various carpet densities.

A statistical model was applied to carpet thickness and resilience after some time period of static loading test data and abrasion thickness loss data generated from a Martindale test. The best models for carpet thickness and resilience and abrasion thickness loss were found using DESIGN EXPERT software [93]. Table 8 presents ANOVA for the models explaining carpet thickness, resilience, and abrasion thickness loss. Figure 14 illustrates the relationship between carpet thickness and pile height after a 3-day time period of static loading for various carpet pile densities. Figure 15a–c show the relationship between resilience and pile height after some time period of static loading for various carpet densities. Additionally, Figure 16 illustrates the relationship between carpet thickness loss and pile height after the abrasion test for various carpet densities.

As shown in Table 8 and Figure 14, the regression Equation (14) for carpet thickness (CT) was obtained from the ANOVA table where A is the pile height (mm), B is the density of the carpet (weft ends/10cm), and CT is the thickness of the carpet (mm) after 3 days of static loading. The coefficient of regression (R-squared) was 0.9919 and the mean absolute percent error was 1.28%. The general form of CT Equation (14) obeys the second-order polynomial function where pile height (A) is the main parameter before carpet density (B) of carpet thickness after static loading. The interaction graph of pile height and thickness after a 3-day time period of static loading was also fitted with the regression equations and exhibited in Figure 14.

(14) CT=+7.46638−0.34201×A−0.10486×B+0.022778×A2+8.82395E−004×B2+4.93843E−003×A×B

As shown in Table 8 and Figure 15a–c, the regression Equation (15) for carpet resilience (CR) was obtained from the ANOVA table where A is the pile height (mm), B is the density of the carpet (weft ends/10cm), C is the recovery period (2 min, 1 h, 1 day, and 3 days), and CR is the resilience of the carpet (mm) after static loading. The coefficient of regression (R-squared) was 0.7144 and the mean absolute percent error was 6.19%. The general form of CR Equation (15) obeys the second-order polynomial function where the recovery period (C) is the main parameter before pile height (A) of carpet resilience after static loading. The interaction graph of pile height, density, and resilience after various time periods of static loading was also fitted with the regression equations and illustrated in Figure 15a–c.

(15) $$\begin{array}{l} \mathrm{CR}=+42.22527+11.27528\times A-2.55068\times B+0.49040\times C-0.71824\times A2+0.018216\times B2-\\ \;3.46165E-003\times C2+0.026250\times A\times B-6.42826E-003\times A\times C+4.65152E-004\times B\times C \end{array}$$

As shown in Table 8 and Figure 16, the regression Equation (16) of carpet abrasion thickness loss (CTL) was obtained from the ANOVA table where A is the pile height (mm), B is the density of the carpet (weft ends/10cm), and CTL is the abrasion thickness loss of the carpet (mm) after Martindale abrasion testing. The coefficient of regression (R-squared) was 0.9199 and the mean absolute percent error was 21.71%. The general form of CTL Equation (16) obeys the second-order polynomial function where pile height (A) is the main parameter before carpet density (B) of carpet thickness after abrasion. The interaction graph of pile height and thickness loss on various densities after abrasion was also fitted with the regression equations and exhibited in Figure 16.

(16) CTL=−10.57183−0.30387×A+0.38848 ×B−3.76335E−003× B2+9.56135E−003×A×B

## 4. Conclusions

It was found that the bending rigidity (mN.m) based on the technical fabric test increased from loose to very dense carpet structures, probably due to the large size of the carpet samples in that the size of the sample affects the bending stiffness. Further, the average dry bending rigidity of polyester carpet was over eight times higher than the average wet bending rigidity of the carpet. The bending curvature (cm) increased from loose to very dense carpet structures due to the increase in weft density and the size of the samples. In addition, downward concave small bending curvature was obtained from loose carpet, whereas downward concave large bending curvature was obtained from very dense carpet. In addition, the average dry bending curvature of polyester carpet was higher than the average wet bending curvature of the carpet due to the increase in wet carpet weight. It was concluded that bending through the technical fabric test can simplify the determination of heavy three-dimensional dry or impregnate polymeric preforms’ flexural properties.

The thickness loss (%) and resilience (%) for each recovery period from loose to very dense polyester carpets proportionally depended on the pile density. It generally decreased when the pile density increased due to compression of the load carrying capacity per knot, in which it was higher in carpets having dense knots compared to ones that had sparse knots per area. When the pile density increased, the carpet mass losses (%) of all carpets under abrasion load increased. At the same pile density, when the pile height increased, the carpet mass losses (%) of all carpet samples slightly increased. Additionally, after the number of strokes required to achieve completely fractured pile yarns in the rubbing test increased when the pile heights for each pile density increased.

For future studies, we will carry out more research on particular complex shape molding part manufacturing via generated load–displacement and stress–strain curves.

## Figures and Tables

**Table 1:** Fiber, yarn, and substrate properties of polyester carpet structure.

Sample	Sample Codes	Yarn Linear Density (Tex)			Yarn Composition (%)			Density (Ends/10 cm)		Substrate Weight (Warp + Weft) (g/m^2^)
		Warp		Weft	Warp		Weft	Warp	Weft	
		Stuffer	Chain		Stuffer	Chain				
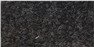	1PES6 1PES9 1PES12	211	118	556	80% Pes/20% Co	80% Pes/20% Co	Jute	48	48	843
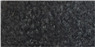	2PES6 2PES9 2PES12	211	118	556	80% Pes/20% Co	80% Pes/20% Co	Jute	48	55	925
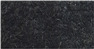	3PES6 3PES9 3PES12	211	118	556	80% Pes/20% Co	80% Pes/20% Co	Jute	48	70	896

**Table 2:** Pile yarn and carpet structure properties.

Sample Codes	Pile						Carpet	
	Fiber Type	Yarn Linear Density (Tex)	Substrate Thickness (mm)	Pile Height (mm)	Pile Density (Knots/m^2^)	Pile Weight (g/m^2^)	Carpet Thickness (mm)	Carpet Weight (g/m^2^)
1PES6	Polyester	165	2.57	6 (3.34)	230,400	954	5.91	1797 (1890)
1PES9	Polyester	165	2.62	9 (4.21)	230,400	1223	6.82	2066 (2142)
1PES12	Polyester	165	2.59	12 (5.88)	230,400	1453	8.47	2297 (2219)
2PES6	Polyester	165	2.63	6 (3.36)	264,000	1049	5.99	1974 (2098)
2PES9	Polyester	165	2.59	9 (4.83)	264,000	1357	7.42	2282 (2121)
2PES12	Polyester	165	2.65	12 (5.88)	264,000	1621	8.53	2546 (2650)
3PES6	Polyester	165	3.05	6 (3.54)	336,000	1253	6.58	2149 (2343)
3PES9	Polyester	165	3.16	9 (4.46)	336,000	1645	7.61	2541 (2477)
3PES12	Polyester	165	3.19	12 (6.46)	336,000	1981	9.65	2877 (2875)

**Table 3:** Flexural results of dry and wet form of polyester carpets.

**Bending for Apparel Fabric**				
**Sample**	**Dry**		**Wet**	
	**Length of Overhang** **l (cm)**	**Bending Rigidity** **G_1_ (mg.cm)**	**Length of Overhang** **l (cm)**	**Bending Rigidity** **G_1_ (mg.cm)**
1PES6-G_1_	14.50 ± 0.08	72,028.90 ± 1216.73	8.48 ± 0.19	14,397.10 ± 946.09
1PES9-G_1_	14.53 ± 0.17	82,075.36 ± 2881.66	8.38 ± 0.34	15,786.35 ± 1873.15
1PES12-G_1_	12.28 ± 0.36	51,400.23 ± 4449.87	7.83 ± 0.25	13,320.29 ± 1263.42
2PES6-G_1_	13.43 ± 0.78	63,929.32 ± 10,601.42	7.93 ± 0.38	13,119.76 ± 1869.14
2PES9-G_1_	14.48 ± 0.21	80,446.04 ± 3440.73	8.20 ± 0.34	14,672.87 ± 1732.53
2PES12-G_1_	10.90 ± 1.42	44,462.45 ± 14,881.99	7.23 ± 0.31	12,544.26 ± 1568.66
3PES6-G_1_	10.33 ± 1.85	34,524.16 ± 16,745.18	6.90 ± 0.50	9735.21 ± 2186.97
3PES9-G_1_	10.73 ± 0.51	38,387.64 ± 5379.77	6.45 ± 0.19	8324.76 ± 730.94
3PES12-G_1_	9.38 ± 2.39	33,774.07 ± 20,862.79	5.68 ± 0.66	6767.60 ± 2376.14
**Bending for technical fabric**				
**Sample**	**Dry**		**Wet**	
	**Length of Overhang** **l (cm)**	**Bending Rigidity** **G_2_ (mN.m)**	**Length of Overhang** **l (cm)**	**Bending Rigidity** **G_2_ (mN.m)**
1PES6-G_2_	18.40 ± 16.26	14.44 ± 3.25	8.60 ± 4.25	1.47 ± 0.22
1PES9-G_2_	19.70 ± 28.99	20.08 ± 6.67	9.30 ± 9.19	2.11 ± 0.49
1PES12-G_2_	20.10 ± 16.97	22.10 ± 3.78	9.60 ± 6.36	2.41 ± 0.22
2PES6-G_2_	19.30 ± 3.54	18.50 ± 0.92	8.90 ± 2.83	1.81 ± 0.30
2PES9-G_2_	20.10 ± 9.90	21.12 ± 2.56	10.40 ± 3.54	2.93 ± 0.20
2PES12-G_2_	20.60 ± 0.71	28.41 ± 0.20	11.10 ± 2.83	4.44 ± 0.26
3PES6-G_2_	21.20 ± 4.95	27.38 ± 0.88	10.10 ± 4.24	2.96 ± 0.18
3PES9-G_2_	22.40 ± 4.24	34.14 ± 0.64	10.60 ± 4.95	3.62 ± 0.26
3PES12-G_2_	22.35 ± 4.95	39.36 ± 0.76	12.00 ± 6.36	6.09 ± 0.25

**Table 4:** Regression equations of bending curvature of dry and wet forms of polyester carpets.

**Bending for Apparel Fabric**				
**Sample Codes**	**Dry**		**Wet**	
	**Equation of** **Regression Curve**	**Coefficient of Regression (R^2^)**	**Equation of** **Regression Curve**	**Coefficient of Regression** **(R^2^)**
1PES6-G_1_	y = 4.404x^0.7361^ − 1.238x + 0.04107	R^2^ = 0.9995	y = 4.553x^0.8316^ − 2.146x + 0.01205	R^2^ = 0.9993
1PES9-G_1_	y = 53.88x^0.9864^ − 51.03x − 0.1173	R^2^ = 0.9993	y = 36.58x^0.9857^ − 34.47x − 0.03946	R^2^ = 0.9989
1PES12-G_1_	y = 36.5x^0.9799^ − 33.72x − 0.08238	R^2^ = 0.9992	y = 15.87x^0.9718^ − 14.02x − 0.01899	R^2^ = 0.9998
2PES6-G_1_	y = − 21.89x^1.029^ + 24.56x + 0.2938	R^2^ = 0.9980	y = 45.24x^0.9906^ − 43.24x − 0.1656	R^2^ = 0.9964
2PES9-G_1_	y = 39.7x^0.9813^ − 36.83x − 0.3762	R^2^ = 0.9954	y = 23.71x^0.9741^ − 21.48x − 0.003374	R^2^ = 0.9999
2PES12-G_1_	y = −26.88x^1.025^ + 29.57x − 0.05452	R^2^ = 0.9989	y = 101.5x^0.9941^ − 99.26x − 0.1659	R^2^ = 0.9902
3PES6-G_1_	y = 5.598x^0.7809^ − 2.351x − 0.03235	R^2^ = 0.9985	y = 5.323x^0.8408^ − 2.866x − 0.008245	R^2^ = 0.9998
3PES9-G_1_	y = 28.67x^0.9749^ − 26.02x − 0.000902	R^2^ = 0.9995	y = 17.04x^0.9506^ − 14.53x − 0.01272	R^2^ = 0.9998
3PES12-G_1_	y = −7.888x^1.072^ + 10.3x − 0.01667	R^2^ = 0.9994	y = −0.3296x^1.763^ + 2.383x − 0.008711	R^2^ = 0.9998
**Bending for technical fabric**				
**Sample Codes**	**Dry**		**Wet**	
	**Equation of** **Regression Curve**	**Coefficient of Regression (R^2^)**	**Equation of** **Regression Curve**	**Coefficient of Regression** **(R^2^)**
1PES6-G_2_	y = 45.62x^0.9883^ − 43.11x − 0.2136	R^2^ = 0.9988	y = 39.03x^0.9894^ − 37.06x − 0.2257	R^2^ = 0.9914
1PES9-G_2_	y = 13.55x^0.9445^ − 10.68x − 0.02309	R^2^ = 0.9997	y = 41.66x^0.9857^ − 39.51x − 0.09046	R^2^ = 0.9968
1PES12-G_2_	y = 4.758x^0.8202^ − 1.804x − 0.03475	R^2^ = 0.9996	y = 59.4x^0.9919^ − 57.18x − 0.2335	R^2^ = 0.9947
2PES6-G_2_	y = 39.37x^0.987^ − 37.02x − 0.1696	R^2^ = 0.9979	y = 58.79x^0.9907^ − 56.82x − 0.1212	R^2^ = 0.9870
2PES9-G_2_	y = 27.29x^0.9814^ − 25.01x − 0.1595	R^2^ = 0.9970	y = 35.46x^0.9804^ − 32.87x − 0.1126	R^2^ = 0.9984
2PES12-G_2_	y = 5.569x^0.8483^ − 2.619x − 0.0268	R^2^ = 0.9997	y = 76.96x^0.9925^ − 74.73x − 0.3564	R^2^ = 0.9524
3PES6-G_2_	y = 28.98x^0.9798^ − 26.31x − 0.2785	R^2^ = 0.9978	y = 25.58x^0.9801^ − 23.53x − 0.09408	R^2^ = 0.9969
3PES9-G_2_	y = 4.663x^0.757^ − 1.286x − 0.02506	R^2^ = 0.9999	y = 59.48x^0.9911^ − 57.49x − 0.2258	R^2^ = 0.9555
3PES12-G_2_	y = 4.675x^0.8484^ − 2.016x − 0.01012	R^2^ = 0.9999	y = 88.58x^0.9898^ − 85.61x − 0.2765	R^2^ = 0.9649

**Table 5:** Recovery period and thickness, thickness loss, and resilience after static loading on various dry carpets.

**Sample Codes**	**Initial Thickness** **(mm)**	**Carpet Thickness of Various Recovery Periods** **(mm)**				
		**h_1_** **(after 2 min.)**	**h_2_** **(after 1 h)**	**h_3_** **(after 1 Day)**	**h_4_** **(3 Days)**	**h_5_** **(3 Years)**
1PES6	5.91	4.21 ± 0.44	4.38 ± 0.28	4.51 ± 0.23	4.62 ± 0.20	5.26 ± 0.79
1PES9	6.82	4.68 ± 0.03	5.16 ± 0.01	5.40 ± 0.02	5.46 ± 0.05	6.27 ± 0.66
1PES12	8.47	5.99 ± 0.05	6.17 ± 0.09	6.37 ± 0.13	6.44 ± 0.12	7.54 ± 0.47
2PES6	5.99	4.22 ± 0.08	4.39 ± 0.11	4.52 ± 0.02	4.76 ± 0.03	5.44 ± 0.54
2PES9	7.42	5.04 ± 0.08	5.31 ± 0.22	5.51 ± 0.18	5.57 ± 0.18	6.14 ± 0.13
2PES12	8.53	6.29 ± 0.03	6.41 ± 0.04	6.66 ± 0.02	6.82 ± 0.01	7.03 ± 0.64
3PES6	6.58	4.98 ± 0.04	5.12 ± 0.04	5.25 ± 0.04	5.34 ± 0.05	5.72 ± 0.20
3PES9	7.61	5.74 ± 0.06	5.93 ± 0.06	6.09 ± 0.04	6.25 ± 0.04	6.66 ± 0.23
3PES12	9.65	7.30 ± 0.07	7.53 ± 0.01	7.70 ± 0.01	7.81 ± 0.01	8.69 ± 0.26
**Sample Codes**	**Initial Thickness** **(mm)**	**Carpet Thickness Loss of Various Recovery Periods** **(%)**				
		**h_1_** **(after 2 min.)**	**h_2_** **(after 1 h)**	**h_3_** **(after 1 day)**	**h_4_** **(3 days)**	**h_5_** **(3 years)**
1PES6	5.91	28.76 ± 7.42	25.89 ± 4.79	23.69 ± 3.83	21.83 ± 3.35	11.00 ± 13.40
1PES9	6.82	31.38 ± 0.41	24.34 ± 0.21	20.89 ± 0.31	20.01 ± 0.73	8.06 ± 9.75
1PES12	8.47	29.34 ± 0.58	27.21 ± 1.09	24.85 ± 1.59	24.03 ± 1.42	11.04 ± 5.59
2PES6	5.99	29.55 ± 1.42	26.79 ± 1.77	24.62 ± 0.35	20.53 ± 0.47	9.18 ± 8.97
2PES9	7.42	32.08 ± 1.14	28.50 ± 2.95	25.81 ± 2.38	24.93 ± 2.48	17.32 ± 1.81
2PES12	8.53	26.26 ± 0.33	24.91 ± 0.41	21.98 ± 0.25	20.05 ± 0.17	17.64 ± 7.54
3PES6	6.58	24.32 ± 0.64	22.19 ± 0.64	20.29 ± 0.54	18.92 ± 0.75	13.07 ± 3.01
3PES9	7.61	24.57 ± 0.74	22.08 ± 0.74	19.97 ± 0.56	17.87 ± 0.56	12.48 ± 2.97
3PES12	9.65	24.35 ± 0.73	22.02 ± 0.07	20.21 ± 0.15	19.07 ± 0.15	10.00 ± 2.71
**Sample Codes**	**Initial Thickness** **(mm)**	**Carpet Resilience of Various Recovery Periods** **(%)**				
		**h_1_** **(after 2 min.)**	**h_2_** **(after 1 h)**	**h_3_** **(after 1 day)**	**h_4_** **(3 days)**	**h_5_** **(3 years)**
1PES6	5.91	-	9.12 ± 6.80	16.59 ± 8.20	23.06 ± 8.20	66.66 ± 37.99
1PES9	6.82	-	22.43 ± 0.36	33.41 ± 1.87	36.21 ± 3.16	74.30 ± 30.72
1PES12	8.47	-	7.24 ± 1.85	15.29 ± 3.72	18.11 ± 3.21	62.37 ± 18.32
2PES6	5.99	-	9.32 ± 1.65	16.67 ± 2.80	30.51 ± 1.74	68.93 ± 28.91
2PES9	7.42	-	11.13 ± 6.05	19.54 ± 4.56	22.27 ± 4.96	46.01 ± 7.57
2PES12	8.53	-	5.13 ± 0.38	16.29 ± 0.11	23.66 ± 1.60	32.81 ± 29.58
3PES6	6.58	-	8.75 ± 0.23	16.56 ± 4.42	22.19 ± 5.16	46.25 ± 10.95
3PES9	7.61	-	10.16 ± 0.31	18.72 ± 0.19	27.27 ± 0.07	49.20 ± 13.64
3PES12	9.65	-	9.57 ± 3.02	17.02 ± 1.90	21.70 ± 2.96	58.94 ± 12.37

**Table 6:** Abrasion cycle, carpet pile mass loss, and carpet thickness loss relations after a Martindale abrasion test on various carpets.

Sample Codes	Abrasion Cycles	Carpet Mass (mg)	Carpet mass Loss (mg)	Carpet mass Loss (%)	Carpet Thickness (mm)	Carpet Thickness Loss (mm)	Carpet Thickness Loss (%)
1PES6	0	2030	0.00 ± 0.00	0.00 ± 0.00	5.91 ± 0.06	0.49 ± 0.14	8.30 ± 2.47
	5000	2015	15 ± 7.07	0.74 ± 0.37	-		
	10,000	2010	20 ± 0.00	0.99 ± 0.03	-		
	20,000	2005	25 ± 7.07	1.23 ± 0.31	-		
	30,000	2005	25 ± 7.07	1.23 ± 0.31	-		
	40,000	1995	35 ± 7.07	1.72 ± 0.30	-		
	50,000	1990	40 ± 0.00	1.97 ± 0.05	5.42 ± 0.20		
1PES9	0	2385	0.00 ± 0.00	0.00 ± 0.00	6.82 ± 0.11	0.57 ± 0.10	9.33 ± 2.70
	5000	2380	5 ± 7.07	0.21 ± 0.30	-		
	10,000	2370	15 ± 7.07	0.63 ± 0.29	-		
	20,000	2360	25 ± 21.21	1.05 ± 0.89	-		
	30,000	2350	35 ± 21.21	1.47 ± 0.89	-		
	40,000	2340	45 ± 21.21	1.89 ± 0.88	-		
	50,000	2335	50 ± 14.41	2.10 ± 0.59	6.25 ± 0.01		
1PES12	0	2530	0.00 ± 0.00	0.00 ± 0.00	8.47 ± 0.07	1.34 ± 0.17	15.81 ± 1.87
	5000	2525	5 ± 7.07	0.20 ± 0.28	-		
	10,000	2515	15 ± 7.07	0.59 ± 0.28	-		
	20,000	2505	25 ± 7.07	0.99 ± 0.29	-		
	30,000	2485	45 ± 7.07	1.78 ± 0.27	-		
	40,000	2465	65 ± 7.07	2.57 ± 0.29	-		
	50,000	2455	75 ± 7.07	2.97 ± 0.30	7.13 ± 0.10		
2PES6	0	2260	0.00 ± 0.00	0.00 ± 0.00	5.99 ± 0.04	0.59 ± 0.11	9.86 ± 1.95
	5000	2225	35 ± 21.21	1.56 ± 0.97	-		
	10,000	2175	85 ± 7.07	3.76 ± 0.24	-		
	20,000	2160	100 ± 0.00	4.43 ± 0.08	-		
	30,000	2150	110 ± 0.00	4.87 ± 0.09	-		
	40,000	2145	115 ± 7.07	5.09 ± 0.41	-		
	50,000	2130	130 ± 0.00	5.75 ± 0.11	5.40 ± 0.15		
2PES9	0	2110	0.00 ± 0.00	0.00 ± 0.00	7.42 ± 0.12	1.58 ± 0.04	21.32 ± 0.92
	5000	2090	20 ± 0.00	0.95 ± 0.07	-		
	10,000	2045	65 ± 35.36	3.03 ± 1.45	-		
	20,000	2025	85 ± 21.21	4.00 ± 0.71	-		
	30,000	2015	95 ± 21.21	4.48 ± 0.68	-		
	40,000	1975	135 ± 77.78	6.28 ± 3.22	-		
	50,000	1920	190 ± 141.42	8.78 ± 6.05	5.84 ± 0.16		
2PES12	0	2665	0.00 ± 0.00	0.00 ± 0.00	8.53 ± 0.28	2.05 ± 0.42	23.91 ± 4.10
	5000	2655	10 ± 0.00	0.38 ± 0.00	-		
	10,000	2650	15 ± 7.07	0.56 ± 0.27	-		
	20,000	2615	50 ± 14.14	1.87 ± 0.51	-		
	30,000	2585	80 ± 28.28	3.00 ± 1.02	-		
	40,000	2555	110 ± 0.00	4.13 ± 0.05	-		
	50,000	2345	320 ± 84.85	11.99 ± 3.02	6.49 ± 0.13		
3PES6	0	2675	0.00 ± 0.00	0.00 ± 0.00	6.58 ± 0.10	0.24 ± 0.08	3.64 ± 1.23
	5000	2665	10 ± 0.00	0.37 ± 0	-		
	10,000	2660	15 ± 7.07	0.56 ± 0.26	-		
	20,000	2650	25 ± 7.07	0.93 ± 0.26	-		
	30,000	2550	125 ± 91.92	4.69 ± 3.47	-		
	40,000	2535	140 ± 113.14	5.25 ± 4.27	-		
	50,000	2525	150 ± 113.14	5.62 ± 4.27	6.34 ± 0.01		
3PES9	0	2715	0.00 ± 0.00	0.00 ± 0.00	7.61 ± 0.10	1.78 ± 0.36	23.30 ± 4.44
	5000	2690	25 ± 7.07	0.92 ± 0.27	-		
	10,000	2440	275 ± 35.36	10.12 ± 1.22	-		
	20,000	2420	295 ± 35.36	10.86 ± 1.22	-		
	30,000	2410	305 ± 35.36	11.23 ± 1.21	-		
	40,000	2395	320 ± 42.43	11.78 ± 1.47	-		
	50,000	2365	350 ± 70.71	12.88 ± 2.50	5.84 ± 0.26		
3PES12	0	2860	0.00 ± 0.00	0.00 ± 0.00	9.65 ± 0.13	2.39 ± 0.10	24.79 ± 1.37
	5000	2850	10 ± 0.00	0.35 ± 0.00	-		
	10,000	2835	25 ± 7.07	0.87 ± 0.25	-		
	20,000	2820	40 ± 14.14	1.40 ± 0.50	-		
	30,000	2795	65 ± 21.21	2.27 ± 0.73	-		
	40,000	2770	90 ± 56.57	3.14 ± 1.96	-		
	50,000	2740	120 ± 98.99	4.19 ± 3.44	7.26 ± 0.23		

**Table 7:** Stroke number and fractured pile yarn relations after token rubbing test on various carpet samples.

Sample Codes	Stroke Number at the Beginning of Fractured Pile Yarns	Stroke Number after Complete Fractured Pile Yarns
1PES6	1125	11,000
1PES9	1250	19,750
1PES12	1750	23,750
2PES6	1750	14,750
2PES9	1750	20,000
2PES12	2000	32,250
3PES6	2250	7000
3PES9	1750	18,250
3PES12	1750	23,000

**Table 8:** ANOVA tables for the model of carpet thickness and resilience after static loading and abrasion thickness loss.

**Carpet Thickness**							
**Source**	**Sum of Squares**	**Contribution (%)**	**DF**	**Mean Square**	**F Value**	**Prob > F**	**Significance**
Model	16.89	99.19	5	3.38	293.23	<0.0001	significant
A	13.66	80.21	1	13.66	1185.60	<0.0001	significant
B	2.77	16.29	1	2.77	240.82	<0.0001	significant
A^2^	0.17	0.99	1	0.17	14.59	0.0024	significant
B^2^	0.03	0.19	1	0.03	2.85	0.1169	
AB	0.22	1.30	1	0.22	19.26	0.0009	significant
Residual	0.14	0.81	12	0.01			
Lack of Fit	0.04	0.25	3	0.01	1.35	0.3188	not significant
Pure Error	0.10	0.56	9	0.01			
Cor Total	17.03	100.00	17				
**Resilience**							
**Source**	**Sum of Squares**	**Contribution (%)**	**DF**	**Mean Square**	**F Value**	**Prob > F**	**Significance**
Model	2646.52	71.44	9	294.06	12.23	<0.0001	significant
A	35.75	0.97	1	35.75	1.49	0.2292	
B	93.52	2.52	1	93.52	3.89	0.0549	
C	1910.68	51.58	1	1910.68	79.46	<0.0001	significant
A^2^	501.43	13.54	1	501.43	20.85	<0.0001	significant
B^2^	42.05	1.14	1	42.05	1.75	0.1929	
C^2^	168.31	4.54	1	168.31	7.00	0.0113	significant
AB	18.80	0.51	1	18.80	0.78	0.3813	
AC	11.71	0.32	1	11.71	0.49	0.4889	
BC	0.86	0.02	1	0.86	0.04	0.8508	
Residual	1058.04	28.56	44	24.05			
Lack of Fit	669.36	18.07	17	39.37	2.74	0.0095	significant
Pure Error	388.67	10.49	27	14.40			
Cor Total	3704.55	100.00	53				
**Abrasion thickness loss**							
**Source**	**Sum of Squares**	**Contribution (%)**	**DF**	**Mean Square**	**F Value**	**Prob > F**	**Significance**
Model	9.09	91.99	4	2.27	37.31	<0.0001	significant
A	7.16	72.47	1	7.16	117.57	<0.0001	significant
B	1.35	13.62	1	1.35	22.10	0.0004	significant
B^2^	0.60	6.05	1	0.60	9.82	0.0079	
AB	0.83	8.41	1	0.83	13.65	0.0027	significant
Residual	0.79	8.01	13	0.06			
Lack of Fit	0.55	5.60	4	0.14	5.21	0.0188	significant
Pure Error	0.24	2.42	9	0.03			
Cor Total	9.88	100.00	17				

## Data Availability

Data are provided in the article.
